# Maternal and child-health outcomes in pregnancies following Assisted Reproductive Technology (ART): a prospective cohort study

**DOI:** 10.1186/s12884-020-2755-z

**Published:** 2020-02-21

**Authors:** Shana Ginar da Silva, Mariângela Freitas da Silveira, Andréa Dâmaso Bertoldi, Marlos Rodrigues Domingues, Iná da Silva dos Santos

**Affiliations:** 1 Postgraduate Program in Epidemiology, Federal University of Pelotas, Rua Marechal Deodoro, 1160 - 3° piso, CEP: 96020-220, Bairro Centro, Pelotas, Rio Grande do Sul Brazil; 2 Medical school, Federal University of Fronteira Sul - Campus Passo Fundo, RS. Rua Cap. Araújo, 20 - Passo Fundo, Pelotas, Rio Grande do Sul 99010-121 Brazil; 3 Postgraduate Program in Physical Education, Federal University of Pelotas, R. Luís de Camões, 625 - Três Vendas, Pelotas, RS 96055-630 Brazil

**Keywords:** Assisted reproductive technology, Perinatal health, In vitro fertilization, Neonatal outcomes, Maternal-child health

## Abstract

**Background:**

Studies comparing the outcome of spontaneous versus assisted reproductive technologies (ART) pregnancies report heterogeneous results. Despite the success of ART to overcome infertility, concern is growing regarding both its safety and its effect on maternal and child health. The objective of this study was to compare maternal and child-health outcomes after ART relative to natural conception.

**Methods:**

A population-based birth cohort study was carried out among pregnant women expected to deliver in 2015 in Pelotas, southern Brazil. Maternal outcomes included pregnancy complications and gestational weight gain. Gestational age, weight, intrauterine growth restriction, length and head circumference, and 1-min and 5-min Apgar, as well as health problems at birth and breastfeeding were defined as offspring outcomes. Statistical analyses were performed using linear and logistic regression. G-formula was used to perform mediation analysis.

**Results:**

The study included 4252 babies born by spontaneously pregnancies and 23 babies born after ART. Adjusted analyses showed that children conceived from ART presented lower means of gestational age (*p* = 0.001), birth weight (*p* = 0.002), length (*p* < 0.001), and head circumference at birth (*p* = 0.02). However, more than 90% of the effect of ART over these outcomes was mediated by multiple pregnancy.

**Conclusion:**

Our findings suggest that the possible negative effect on the child-health outcomes is due mainly to the higher incidence of multiple pregnancies and not because of ART. The reasons for the increase in adverse pregnancy outcomes associated with ART singleton pregnancies are still uncertain and warrants further research. Further large-population studies are needed to confirm these results.

## Background

As a result of advanced technologies and the number of fertility services providing assisted reproductive technology (ART), an increasing number of infants are born as a result of ART procedures worldwide [2, 7]. In high-income countries, ART pregnancies represent 1.5 to 5.9% of all births [5, 6, 21, 29]. Despite the success of ART to overcome infertility, concern is growing regarding both its safety and its effect on maternal and child health.

Previous studies reported that pregnancies following ART have higher risk of adverse neonatal outcomes, including preterm delivery, low birth weight, birth defects, and perinatal mortality [12, 14, 15, 29, 33] when compared to spontaneous conception, even when limited to singleton births [15, 29]. Data from two meta-analyses have confirmed that ART involving in vitro fertilization (IVF) and/or intracytoplasmic sperm injection (ICSI) was associated with increased risk of adverse perinatal outcomes [15, 25]. Some studies have suggested increased risk for maternal health as well, like pregnancy-related hypertensive complications [15, 30], gestational diabetes [15] and other maternal morbidity [23]. On the other hand, some studies have reported that the ART procedures associated with IVF and ICSI are not responsible for those adverse health-related outcomes. This hypothesis is supported by studies of subfertile women who conceived without ART and yet showed increased risk of adverse maternal and neonatal outcomes [18, 22]. Several studies have been conducted to address this issue, but their results are often inconsistent.

Potential reasons for the poorer perinatal health of ART-mothers and children has been related to procedures involving ART, such as medicines used, altered hormonal environment at the time of implantation, and the manipulation of gametes and embryos or a combination of these [11, 12, 16]. It has also been found that the risk for preterm birth increases with ART low-technology treatments, compared with natural pregnancy, and increases further with ART high-technology treatments ([32]). However, there is scarce data examining the type of ART used in relation to the maternal and live-birth outcomes and the results available are controversial.

It has also been suggested that the worse health in infants born is due mainly to the higher risk incidence of multiple pregnancies and not related to ART procedures [26, 34]. On the whole, most of previous estimates of potential adverse health outcomes among ART pregnancies have been based in studies from high-income countries. As far as we are aware, none study on the effect of ART on maternal-child health have been conducted in low-and-middle income countries such as Brazil. Also, many prior studies failed to control for maternal age and other relevant potential confounders as gravidity and parity history. It remains a challenge to infertility research.

To better understand the impact of ART on maternal and child health, we compared maternal and child health outcomes after ART technologies relative to natural conception in a birth cohort study in southern Brazil.

## Methods

### Study design

We analyzed data from a population-based birth cohort study designed to include pregnant women living in the urban area of Pelotas, Southern Brazil, with an expected delivery date from January 1st, 2015 to 31st December 2015. The Pelotas 2015 Birth Cohort Study recruited pregnant women from all health facilities offering antenatal care including public and private services. During antenatal phase, three types of questionnaires were applied according to the gestational age of the woman at enrolment. Those before 16 weeks of pregnancy answered the initial assessment questionnaire and were contacted again between 16 and 24 weeks to answer the main assessment questionnaire. Women who were enrolled after 16 weeks of gestation answered to a combined assessment questionnaire containing questions from the *initial* and the *main assessments.* Thereafter, mothers were interviewed at hospital up to 48 h after delivery (perinatal study). Details on the cohort design, recruitment and enrollment can be found elsewhere [10].

The study was approved by the School of Physical Education Ethics Committee of the Federal University of Pelotas in an official letter numbered 522/064. Written informed consent was obtained from all participants.

### ART exposure

Utilization of an ART procedure was gathered at the antenatal and the perinatal interviews. Women who answered *“yes”* to the question:*” Did you have an artificial fertilization in this pregnancy?* were contacted later (during 2017) and were invited to take part in a sub-study. Women in the sub-study were interviewed by phone. After five failed attempts of a phone interview, women were searched and interviewed at home.

Utilization of an ART procedure was firstly confirmed and then other related characteristics, such as type of ART were investigated. ART procedures included in vitro fertilization (IVF), intracytoplasmic sperm injection (ICSI) and artificial insemination (AI). Conceptually, ART does not include assisted insemination (artificial insemination) using sperm from either the woman’s partner or a sperm donor. However, considering that women who performed this procedure did not have a natural conception, we decided to include artificial insemination as part of our exposure.

### Outcomes

Maternal health outcomes included mode of delivery (cesarean/vaginal); pregnancy complications (arterial hypertension, pre-eclampsia, gestational diabetes, and hospitalization during pregnancy) reported by the mother or extracted from medical records at the mother’s prenatal care card; and number of offspring delivered (single or multiple pregnancy).

Gestational weight gain was analyzed in two ways: (I) weight at the end of pregnancy (as recorded at the maternal prenatal care card or reported by the mother) minus the reported pre-pregnancy weight (II) assessed following the 2009 Institute of Medicine recommendations based on pre-pregnancy BMI and total gestational weight gain reported by the mothers at the hospital after delivery. Recommended weight gains during pregnancy for underweight, normal weight, overweight, and obese women were 12.5 to 18 kg, 11.5 to 16 kg, 7 to 11.5 kg, and 5 to 9 kg, respectively. Maternal weight at 3 months postpartum (kg) was collected by the research team with a TANITA scale model UM-080 with precision of 100 g and capacity for up to 150 kg.

Child health outcomes included gestational age at delivery that was calculated based on information collected in antenatal and perinatal study as follows: data on the last menstrual period as recorded at the woman’s prenatal care card and/or by maternal self-report; and through the obstetric ultrasound performed before 16 weeks of pregnancy. The final variable of gestational age was estimated by an algorithm that considered all the information previously collected, as well as the plausibility of the estimates based on birth weight, length and head circumference, according to the Fetal and Neonatal Growth Curves for the twenty-first Century [8]. Newborns with gestational age < 37 weeks were categorized as preterm. Intrauterine growth restriction (weight < 10 percentile for gestational age) and health problems at birth were also investigated.

Birth weight and 1-min and 5-min Apgar were collected from medical records at the hospital. Length and head circumference at birth were measured at the hospital up to 48 h after delivery by the trained research team. Information on health problems at birth, breastfeeding duration and breastfeeding up to 12 months after birth was obtained at the 12-month follow-up.

### Covariables

Family income (1–3; 3.1–10; > 10 minimum wages) (01 minimum wage was equivalent to US$ 300.00) and maternal age (< 30; 30–35; 36–40; > 40 years), parity (1; ≥ 2), schooling (0–8; 9–11; ≥ 12 completed years), and pre-pregnancy body mass index (BMI) were used as independent covariables. Pre-pregnancy BMI was calculated dividing weight by height squared. Pre-pregnancy maternal weight and height were self-reported. Women were classified as underweight (< 18.5 kg/m^2^), normal weight (18.5–24.9 kg/m^2^), overweight (25.0–30.0 kg/m^2^), and obese (> 30 kg/m^2^), according to cutoffs defined by the World Health Organization.

### Statistical analysis

Between groups difference was analyzed using the Student’s t-test (mean, SD) for continuous variables or the chi-squared and Fisher’s Exact test for categorical variables (n, %). Normality of data were checked graphically using histograms and by the parameters mean, median, skewness, and kurtosis. All continuous outcomes presented symmetric distribution.

Unadjusted and adjusted analyses were performed using linear regression expressed in β coefficients or binomial logistic regression expressed in odds ratios (OR). Analyses were adjusted for family income, maternal age, parity, and pre-pregnancy BMI. Mediation analyses were performed for statistically significant associations using G-computation in order to verify the proportion of the association between ART and child-health outcomes captured by multiple pregnancies. This was done by estimating the natural direct effects (NDE) and natural indirect effects (NIE) of ART on child-health outcomes. The NDE represents the effect of the exposure (ART) on the outcomes (gestational age at delivery, birth weight, length at birth and head circumference at birth) that is not captured by the mediator (multiple pregnancies), while the NIE estimates the effect that is captured. Therefore, the sum of the NDE and NIE would represent the total effect, and the quotient of dividing the NIE by the total effect would represent the percentage of the effect that is captured by a mediator. In our mediation analyses maternal age, family income, maternal schooling, parity, and pre-pregnancy BMI were considered as base-confounders and hospitalization during pregnancy as a post-confounder.

Statistical significance was set at 5, and 95% confidence intervals were adopted. All the analyses were performed using the software Stata version 12.1 (StataCorp, College Station, Texas, US).

## Results

Out of the 4333 live births that took place in Pelotas in 2015 to mothers living in the urban area of the city, 4275 newborns were enrolled in the 2015 Birth Cohort Study (~ 1.3% losses and refusals) and 4219 were entered in the current analyses. Of these, eighteen mothers gave birth to 23 newborns (0.4%) conceived by ART. The ART group was comprised of the following techniques: in vitro fertilization (*n* = 12), intracytoplasmic sperm injection (*n* = 2) and artificial insemination (*n* = 3). Only one of the 18 ART-mothers refused to inform the ART technique.

Table 1 presents prevalence of pregnancies conceived after ART according to family income and maternal characteristics. Prevalence of ART was higher among mothers from higher socioeconomic position (*p* < 0.001), older (*p* < 0.001), primiparae (*p* = 0.001), and that had more years of schooling (*p* < 0.001).

**Table 1:** Prevalence of pregnancy by assisted reproductive technology (ART) according to maternal characteristics. The 2015 Pelotas (Brazil) Birth Cohort Study

	2015 Birth Cohort	ART		*p*^a^
	N	n	%	
Family income				< 0.001
1–3	2525	4	0.2	
3.1–10	1430	5	0.3	
> 10	262	9	3.4	
Age (years)				< 0.001
< 30	2613	2	0.1	
30–35	1130	8	0.7	
36–39	350	5	1.4	
≥ 40	125	3	2.4	
Parity				0.001
1 (primiparae)	2084	16	0.8	
≥ 2	2133	2	0.1	
Schooling (years)				< 0.001
0–8	1471	1	0.1	
9–11	1441	1	0.1	
12+	1305	16	1.2	
Pre-pregnancy BMI ≥ 25 (kg/m^2^)	2000	8	0.7	0.60
Total	4219	18	0.4	

^a^Fisher’s exact test; *ART* assisted reproductive technology, *BMI* body mass index

Incidence of maternal health outcomes in pregnancies after ART, as compared to spontaneous pregnancies are shown in Table 2. There was higher rate of multiple pregnancies in ART-mothers than in mothers from spontaneous pregnancies (22.2% vs. 1.2%, respectively; *p* < 0.001). All women who conceived after ART had their babies by cesarean section. No significant differences were found regarding occurrence of maternal pregnancy complications, gestational weight gain or body weight at 3 months postpartum (Table 2).

**Table 2:** Incidence of maternal health outcomes from spontaneous pregnancies and from pregnancies by assisted reproductive technology (ART). The 2015 Pelotas (Brazil) Birth Cohort Study

	Spontaneous pregnancy (*n* = 4201)		ART (*n* = 18)		
	***n***	**%**	***n***	**%**	***p***^**a**^
Multiple pregnancies					< 0.001*
Yes	51	1.2	4	22.2	
No	4150	98.8	14	77.8	
Mode of delivery					0.001*
Cesarean	2717	64.7	18	100.0	
Vaginal	1483	35.3	0	0.0	
Pregnancy complications					
Arterial hypertension	1066	25.4	2	11.1	0.27
Pre-eclampsia	266	6.4	1	5.6	1.00
Gestational diabetes	358	8.5	3	16.7	0.20
Hospitalization	829	19.7	4	22.2	0.79
Weight gain during pregnancy					0.91
Bellow IOM recommendations	1185	30.5	6	35.3	
Within IOM recommendations	1261	32.4	5	29.4	
Exceeded IOM recommendations	1446	37.2	6	35.3	
	**Mean**	**DP**	**Mean**	**DP**	***p***^***b***^
Gestational weight gain (kg)	12.0	6.6	11.6	5.1	0.80
Weight at three months postpartum (kg)	69.6	15.3	69.5	12.8	0.97

^a^Fisher’s exact test, ^b^Student T-test. *ART* assisted reproductive technology, *BMI* body mass index. *p < 0.05

Child- health outcomes from spontaneous pregnancies and ART are presented in Table 3. When singleton and multiple pregnancies were analyzed together (total births), neonates born after ART techniques had lower birth weight (2832 ± 6.99 g vs. 3.171 ± 5.63 g) and shorter birth length (46 ± 3.3 cm vs. 48 ± 2.7 cm) than those born from spontaneous pregnancies. Prevalence of preterm births was twice as high among newborns from ART techniques than among those from spontaneous pregnancies (34.8% vs. 15.4%, respectively; *p* = 0.001). On the other hand, when the analyses were stratified by singleton and multiple pregnancies, these associations were not confirmed (Table 3).

**Table 3:** Child- health outcomes from spontaneous pregnancy and assisted reproductive technology (ART). The 2015 Pelotas (Brazil) Birth Cohort Study

	Total pregnancies					Singleton pregnancies					Multiple pregnancies				
	Spontaneous *n* = 4252		ART *n* = 23			Spontaneous *n* = 4150		ART *n* = 14			Spontaneous *n* = 102		ART *n* = 9		
	**mean**	**SD**	**mean**	**SD**	***p***^**a**^	**mean**	**SD**	**mean**	**SD**	***p***^**a**^	**mean**	**SD**	**mean**	**SD**	***p***^**a**^
Gestational age at delivery (weeks)	38.5	2.3	36.9	2.0	0.001*	38.6	2.2	37.9	1.5	0.25	34.2	3.0	35.3	1.8	0.30
1-min Apgar	8.4	1.6	8.4	2.0	0.83	8.4	1.6	9.1	0.9	0.08	7.2	2.1	7.3	2.7	0.88
5-min Apgar	9.4	0.9	9.4	0.9	0.94	9.5	0.9	9.7	0.5	0.28	8.7	1.7	9.0	1.2	0.55
Birthweight (g)	3.171	563	2.832	699	0.004*	3.197	537	3.251	474	0.71	2.070	531	2.178	438	0.55
Length at birth (cm)	48	2.7	46	3.3	< 0.001*	48	2.6	48	1.9	0.71	43	3.4	43	2.4	0.71
Head circumference at birth (cm)	34	1.9	33	2.3	0.06	34	1.9	34	1.4	0.32	31	2.1	31	1.7	0.95
Breastfeeding up to 12 months (days)	223	141	227	151	0.88	226	141	300	106	0.05	96	93.4	114	144	0.60
	**n**	**%**	**n**	**%**	**p**^**b**^	**n**	**%**	**n**	**%**	**p**^**b**^	**n**	**%**	**n**	**%**	**p**^**b**^
Preterm birth (< 37 weeks)	655	15.4	8	34.8	0.01*	574	13.8	2	14.3	1.00	81	79.4	6	66.7	0.40
Breastfeeding at 12 months	1692	42.4	11	47.8	0.60	1687	43.2	9	64.3	0.18	5	5.6	2	22.2	0.12
Intrauterine growth restriction (IUGR)	644	15.3	5	21.7	0.38	611	14.8	1	7.1	0.42	33	34.2	4	44.4	0.72
Health complications at birth	458	10.8	5	21.7	0.09	412	10.0	1	7.1	0.73	46	46.9	4	44.4	0.89

^a^Student’s T-test. ^b^Fisher’s exact test. *ART* assisted reproductive technology, *BMI* body mass index. *p < 0.05

Table 4 displays the results from crude and adjusted linear and binomial logistic regression models. Adjusted analysis showed that babies conceived after ART-techniques presented lower means of gestational age (*p* = 0.001), birth weight (*p* = 0.002) and length at birth (*p* < 0.001), compared to babies born from spontaneous pregnancies. Neonates born after ART also presented higher risk to be preterm (OR: 3.28; 95%CI: 1.32; 8.13) relative to natural conception. Babies conceived after ART had lower means of head circumference at birth (*p* = 0.02).

**Table 4:** Unadjusted and adjusted association between child-health outcomes and ART at the 2015 Pelotas, Brazil, Birth Cohort

	Total pregnancies					
	Crude			Adjusted^a^		
	**β**	**95%CI**	***p***	**β**	**95%CI**	***p***
Gestational age at delivery (weeks)	−1.59	−2.6; −0.64	0.001*	−1.42	−2.29; −0.55	0.001*
1-min Apgar	0.07	−0.59; 0.74	0.83	−0.34	−0.98; 0.30	0.30
5-min Apgar	−0.01	−0.38; 0.35	0.94	−0.18	− 0.51; 0.15	0.28
Birthweight (g)	− 340.13	− 571.19; − 109.07	0.004*	−346.9	− 567.9; − 125.8	0.002*
Length at birth (cm)	−2.17	−3.3; −1.05	< 0.001*	− 2.15	− 3.22; − 1.08	< 0.001*
Head circumference at birth (cm)	−0.75	−1.55; 0.05	0.06	−0.89	−1.66; 0.12	0.02*
Breastfeeding up to 12 months (days)	4.52	− 53.3; 62.4	0.88	−19.0	−76.79; 38.7	0.52
	**OR**	**95%CI**	***p***	**OR**	**95%CI**	***p***
Preterm birth (< 37 weeks)	2.93	1.24; 6.94	0.02*	3.28	1.32; 8.13	0.01*
Breastfeeding at 12 months	1.25	0.55; 2.83	0.60	1.00	0.99; 1.00	0.38
Intrauterine growth restriction (IUGR)	1.54	0.57; 4.17	0.39	1.77	0.63; 4.94	0.28
Health complications at birth	2.29	0.85; 6.21	0.10	2.01	0.72; 5.69	0.18

*CI* confidence interval, *OR* odds ratio, *ART* assisted reproductive technology

^a^Adjusted for maternal age, family income, parity and pre-gestational BMI. *p < 0.05

Mediation analyses showed that all the associations observed between ART and the child-health outcomes measured at birth were mediated by multiple pregnancies. Multiple pregnancies captured 93% of the association between ART and gestational age at delivery. Moreover, multiple pregnancies were responsible for 92 and 94% of the association of ART and birth weight and length, respectively. The percentage of effect on ART mediated by multiple pregnancies was 95% when considered head circumference (Table 5).

**Table 5:** Mediation analysis of the association between ART and child-health outcomes mediated by multiple pregnancies

G-Computation estimate	Total effect β (95%CI)	*p*	Natural direct effect β (95%CI)	*p*	Natural indirect effect β (95%CI)	*p*	Mediated effect (%)
Gestational age at delivery (weeks)	−1.29 (−2.22; −0.38)	0.006*	−0.10 (−0.82; 0.63)	0.79	−1.20 (−2.04; − 0.36)	0.005*	92%
Birthweight (g)	− 302.2 (− 593.7; − 10.7)	0.04*	27.8 (− 145.6; 201.2)	0.75	− 330.1 (−571.3; −88.8)	0.007*	92%
Length at birth (cm)	− 0.82 (− 1.79; 016)	0.10	0.06 (− 0.59; 0.71)	0.85	− 0.88 (− 1.50; − 0.26)	0.006*	94%
Head circumference at birth (cm)	− 0.82 (− 1.78; 0.15)	0.10	0.05 (− 0.60; 0.70)	0.88	− 0.87 (− 1.49; − 0.24)	0.006*	95%

*CI* confidence interval. *p < 0.05

*Adjusted for base confounders maternal age, family income, maternal schooling, parity and pre-pregnancy body mass index and for post confounder hospitalization during pregnancy

Because conceptually ART does not include assisted insemination (artificial insemination) using sperm from either the woman’s partner or a sperm donor, we repeated the analyses after removing cases of artificial insemination and no changes in the results were observed.

## Discussion

Although ART procedures have helped many women to overcome human infertility, concern is growing regarding the possible negative effects of these procedures on maternal and child health. In this paper, we have compared maternal and neonatal outcomes after ART technologies relative to natural conception in a birth cohort study in southern Brazil. Initial analysis showed that babies conceived after ART techniques presented greater risk of adverse health-related outcomes in early life compared to babies born from a spontaneous pregnancy. However, when we performed mediation analyses we observed that the possible negative effect on the child-health outcomes is due mainly to the higher incidence of multiple pregnancies and not because ART techniques.

The literature has presented inconsistent results regarding the effects of ART techniques on maternal and child health. Several studies have been performed to address this issue. While some studies have reported that the ART pregnancies compared with those conceived naturally, whether singleton or multiple, have increased risk of maternal complications [15, 23, 30] and higher risk of preterm birth, low birth weight, birth defects, malformations and perinatal mortality [12, 29], in contrast, other studies have shown that these outcomes are similar between ART and spontaneous conception [18, 22, 31]. In a study with 223 twin pregnancies (84 conceived by IVF and 139 spontaneously conceived) no significant differences were observed on gestational age at delivery, birthweight, perinatal morbidity and mortality, and rate of malformations [31]. In our study any of the unfavorable health-related maternal outcomes (except multiple pregnancies and C-sections) were more incident among the ART group, and child unfavorable health-related outcomes in the ART group was mediated by multiple pregnancy.

The heterogenous results reported so far can perhaps arise from differences in the studied populations and/or in the management approach to twin pregnancies. As part of procedures involving ART, the use of human menopausal gonadotropin during ovarian stimulation has been associated with increases in insulin-like growth factor–binding protein. Evidence have suggested that this protein has been linked to intrauterine growth restriction and preterm birth and low birth weight [17]. However, our findings showed that the preterm birth and low birth weight were probably consequence of multiple pregnancies and not of ART-techniques. A less direct mechanism has also been postulated because women who have conceived after ART are more likely to undergo elective cesarean section, resulting in deliveries that occur earlier than those following spontaneous pregnancies [13]. We did not find differences between complications of pregnancy, but we observed a higher rate of cesarean delivery among ART-mothers. However, this finding may be due to the socio-economic position of mothers who underwent ART procedures. In Brazil there is a strong link between high maternal family income (as is the case of the mothers in the ART group in our study) and cesarean delivery [3].

One of the consequences of the ART-techniques is the progressive rise in the incidence of the twin, triplet and multiples pregnancies. In our study, we observed higher rate of multiple pregnancies in ART-mothers. Although success with ART treatment is surely associated with the number of embryos transferred, higher number of embryo transferred has been strongly associated with adverse perinatal outcomes such as preterm birth and low birth weight [19]. The transfer of more than one embryo is particularly frequent where ART treatments are quite expensive, and couples wish to maximize the chances of getting a pregnancy. However, the final effect is a higher number of multiple pregnancies in ART-mothers. It is well known that neonatal complications are more frequent in twin, triplet and multiple pregnancies than singleton pregnancies [1]. Compared with singleton pregnancies, twin and triplet pregnancies are associated with an increase in fetal, infant, and maternal morbidity and mortality. Infant morbidity includes an increased risk of intrauterine growth restriction and preterm delivery with the associated complications of prematurity. Maternal morbidity includes an increased risk of hypertension, gestational diabetes, hemorrhage, and cesarean delivery [4, 9]. Our results support single-embryo transfer to minimize the risks associated with multiple pregnancies.

Similar to our findings, studies with larger sample sizes (conception by ART “*n*  =  2256” and spontaneous conception “*n*  =  6768”) have demonstrated that patients who underwent ART were at increased risk for several adverse pregnancy outcomes compared to women who conceived spontaneously. These complications were attributed in part to the relatively higher multiple pregnancy rate after ART [20]. These findings were consistent with a prospective cohort study by Moini et al., suggesting that twin pregnancies are indeed an important factor leading to adverse outcomes.

Since 1998 the Society for Assisted Reproductive Technology (SART) and American Society for Reproductive Medicine (ASRM) have published and updated guidelines to assist ART programs and patients in determining the appropriate limit to the number of cleavage-stage embryos or blastocysts to transfer, aiming to promote singleton gestations, reduce twin gestation, and eliminate high-order multiple gestations [28]. A policy of single-embryo transfer in stimulated cycles becomes more popular and is currently the most effective measure to reduce the incidence of multiple pregnancies [24]. With an increasing implementation of a policy of single-transfer embryo, multiple pregnancies have reduced dramatically [27], but is still an outcome of ART that is undesirable.

As far as we are aware, this is the first population-based birth cohort study to assess the effect of ART procedures on maternal-child health in a low-middle income country such as Brazil. In addition, few studies have considered the effect of multiple pregnancies as a mediator on the association between ART and child-health outcomes. However, some limitations such as the small sample size should be mentioned. Our results should be interpreted with caution, especially given the low statistical power to detect the expected outcomes (< 10%) especially in singleton pregnancies. However, it is important to note that the sample size used in our study comes from a large and representative birth cohort study in Brazil. The proportion of ART in the 2015 birth cohort is lower compared to others from high income countries. Reasons for disparities between countries in use of ART procedures may include access barriers such as the high cost of medical services for infertility. Another reason is that governing authorities in low- and middle-income countries face different public health problems, leading them to place lower priority on ART availability. Lack of information on timing to pregnancy in prior pregnancies may be another limitation of our study. Additionally, it has been suggested that the effects of ART on perinatal outcomes may differ according to the techniques applied (IVF vs. ICSI) and etiology of infertility, but our sample size did not allow us to examine these possible differences.

## Conclusions

Mediation analysis showed that the possible negative effect on the child-health outcomes is due mainly to the higher incidence of multiple pregnancies and not because ART-techniques. The reasons for the increase in adverse pregnancy outcomes associated with ART singleton pregnancies are still uncertain and warrants further research. Therefore, further large-population studies are needed to confirm these results. To better understand the impact of ART on maternal-child health, it is important to guide clinicians to inform potential risk of this technology, to provide the basic data for improving medical procedures and consequently reducing the number of transferred embryos. All these aspects would improve the health of ART-mothers and children.

## Data Availability

The dataset supporting of this article are available upon request to the corresponding author.

## Abbreviations

ACOG: The American College of Obstetricians and Gynecologists
AI: Artificial insemination
ART: Assisted reproductive technology
BMI: Body mass index
ICSI: Intracytoplasmic sperm injection
IVF: In vitro fertilization
NDE: Natural direct effects
NIE: Natural indirect effects
